# Therapeutic Potential of PARP Inhibitors in the Treatment of Gastrointestinal Cancers

**DOI:** 10.3390/biomedicines9081024

**Published:** 2021-08-16

**Authors:** Abdullah Alhusaini, Aoife Cannon, Stephen G. Maher, John V. Reynolds, Niamh Lynam-Lennon

**Affiliations:** Department of Surgery, Trinity St. James’s Cancer Institute, Trinity Translational Medicine Institute, St. James’s Hospital, D08 W9RT Dublin 8, Ireland; aalhusai@tcd.ie (A.A.); cannona@tcd.ie (A.C.); maherst@tcd.ie (S.G.M.); reynoljv@tcd.ie (J.V.R.)

**Keywords:** PARP, PARP inhibitors, gastrointestinal cancers, DNA damage response, chemoradiotherapy resistance

## Abstract

Gastrointestinal (GI) malignancies are a major global health burden, with high mortality rates. The identification of novel therapeutic strategies is crucial to improve treatment and survival of patients. The poly (ADP-ribose) polymerase (PARP) enzymes involved in the DNA damage response (DDR) play major roles in the development, progression and treatment response of cancer, with PARP inhibitors (PARPi) currently used in the clinic for breast, ovarian, fallopian, primary peritoneal, pancreatic and prostate cancers with deficiencies in homologous recombination (HR) DNA repair. This article examines the current evidence for the role of the DDR PARP enzymes (PARP1, 2, 3 and 4) in the development, progression and treatment response of GI cancers. Furthermore, we discuss the role of HR status as a predictive biomarker of PARPi efficacy in GI cancer patients and examine the pre-clinical and clinical evidence for PARPi and cytotoxic therapy combination strategies in GI cancer. We also include an analysis of the genomic and transcriptomic landscape of the DDR PARP genes and key HR genes (BRCA1, BRCA2, ATM, RAD51, MRE11, PALB2) in GI patient tumours (*n* = 1744) using publicly available datasets to identify patients that may benefit from PARPi therapeutic approaches.

## 1. Introduction

Gastrointestinal (GI) malignancies (oesophageal, gastric, hepatic, gallbladder, pancreatic and colorectal) are a major global burden, with an incidence of 4.9 million cases per year, and account for 32% of cancer-related mortality worldwide [1]. Conventional GI cancer treatment consists of a multidisciplinary approach including surgery, radiotherapy and pharmacotherapy, which consists of chemotherapy, immunotherapy and targeted therapies (anti-VEGF, anti-EGFR therapies). However, with the currently available treatments, the prognosis of GI cancers remains poor at a mean 5-year survival rate of 27%, ranging from 10–58%, depending on the cancer type [2].

This poor prognosis in GI cancers is a result of a variety of factors, with a subset potentially clinically modifiable, such as delayed diagnosis, body mass index (BMI) variation, and treatment resistance [3,4]. Currently, chemotherapy and radiotherapy are the predominant therapeutics used in most GI cancer types (oesophageal, gastric, hepatic, gallbladder, pancreatic and colorectal cancer). One measure of treatment response is pathological complete response (pCR), defined as the complete absence of residual malignant disease in the primary tumour and lymph node specimens on pathological analysis following surgical resection. A pCR is demonstrated to be associated with improved overall and disease-free survival rates in oesophageal, gastric and rectal tumours [5,6,7,8]. Unfortunately, treatment resistance is a significant clinical problem, with pCR rates in GI cancers ranging from only 0–40% [5,7,8,9,10,11]. Consequently, there is a global unmet clinical need to identify novel treatment strategies to overcome therapy resistance and improve prognosis in GI cancers.

## 2. Poly (ADP-Ribose) Polymerase and the DNA Damage Response (DDR)

Altered DNA damage response (DDR) is an emerging hallmark and enabling characteristic of cancer, associated with both tumour initiation and progression [12,13]. In addition, as anti-cancer cytotoxic agents such as radiation and chemotherapy function to induce DNA damage in cancer cells, alterations in DDR also play a prominent role in resistance to these therapies [14,15,16,17,18,19,20]. This supports the targeting of the DDR to improve treatment and survival of cancer patients. One prominent DDR family of proteins currently being investigated are the poly (ADP-ribose) polymerase (PARP) enzymes [21].

**Figure 1:**
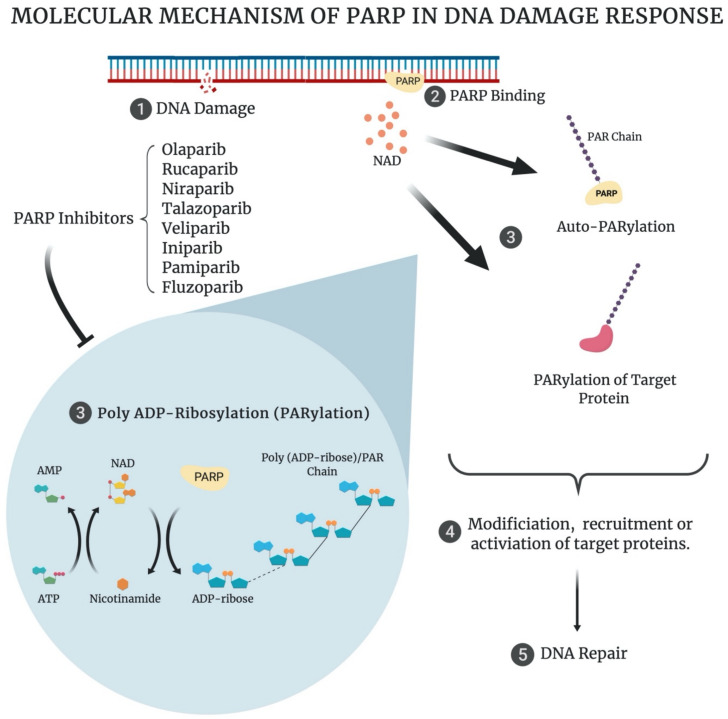
Molecular mechanism of PARP1/2 in the DNA damage response. (**1**). Nuclear DNA is damaged (SSB, DSB) (**2**). PARP1/2 binds to the DNA damage site, activating its enzymatic action. (**3**). PARP1/2 uses an NAD molecule to form an ADP-ribose molecule and a nicotinamide molecule. ADP-ribose is added to the target protein or to existing poly (ADP-ribose) chain. The target protein can be PARP1/2 (Auto-PARylation) or other DDR proteins (PARylation). PARP inhibitors disrupt NAD binding at this step. Nicotinamide is recycled to NAD in the presence of ATP. (**4**). Target DDR proteins are modified, recruited or activated as a result of PARylation. (**5**). Damaged DNA is repaired [32].

The PARP family of enzymes consists of 17 members that can either form poly (ADP-ribose)/PAR chains or mono (ADP-ribose)/MAR chains, from nicotinamide adenine dinucleotide (NAD) molecules, on their target biomolecules (Figure 1). PARP enzymes demonstrate a variety of functions including DNA repair, signal transduction, telomere maintenance, cytoskeletal regulation, transcription regulation, biomolecular transport, antiviral responses and unfolded protein response regulation, among others [22]. Most PARP enzymes have a primary role, with additional overlapping roles with the other PARP enzymes [22]. The four PARP enzymes, PARP1, PARP2, PARP3 and PARP4, are thought to be primarily involved in the DDR.

PARP1 and PARP2 play similar roles in the DDR, with PARP2 activity occurring at a later stage in repair than PARP1 [23]. PARP1 is responsible for 90% of PARP-mediated DNA repair [24]. Both PARP1 and PARP2 are considered sensors of DNA damage, and their binding to DNA in response to damage activates their enzymatic action. This results in the poly (ADP-ribose)-ylation (PARylation), or addition of PAR chains, to their target DDR proteins, resulting in their modification or activation and the recruitment of additional DDR proteins (Figure 1) [25,26]. PARP1 and PARP2 primarily play a role in the first step of single-strand break repair, where they detect single strand breaks (SSB). They auto-PARylate themselves in response to this damage, allowing the recruitment of the X-ray repair cross complementing 1 (XRCC1) protein. XRCC1 acts as a scaffold for the remaining single-strand break repair (SSBR) proteins, allowing SSBR to proceed. In addition, PARP1/2 are key players in the majority of the other DDR pathways, with multiple roles in base excision repair (BER), nucleotide excision repair NER), SSBR, double-strand break (DSB) repair, replication fork repair and chromatin structure modulation [25].

Furthermore, PARP1 plays a role in transcription regulation through ADP-ribosylation-dependent and independent mechanisms. It may also regulate transcription through modulation of chromatin structure, alteration of methylation patterns, acting as a transcription factor co-regulator and interacting with chromatin insulators. This implicates PARP1 in both tumour development, by *ERK2* stimulation resulting in tumour growth, and in tumour progression, through metastasis by PARP1’s pro-inflammatory effects, as a result of its interaction with the NF-κB pathway [27].

PARP3 is predominantly involved in DSB repair, specifically in guiding repair choice (homologous recombination (HR) or non-homologous end joining (NHEJ)) [28,29]. A role for PARP4 in the DDR has not yet been identified; however, it displays a structure similar to the other DDR PARP enzymes and is therefore thought to potentially play a primary role in the DDR [30,31]. As the DDR PARP enzymes are involved in a wide variety of DNA damage repair pathways, their contribution to the genomic stability and, consequently, the survival of cancer cells, support their therapeutic targeting in cancer.

## 3. PARP Inhibitors and ‘Synthetic Lethality’

PARP inhibitors (PARPi) (ending in ‘-parib’) work by binding to the catalytic domain of the PARP enzymes (Figure 1). PARP inhibitors have been demonstrated to bind PARP1 and PARP2 predominantly, as they are structurally very similar, with binding to PARP3 and PARP4 less prominent [33,34]. Catalytic inhibition prevents the many PARylation-mediated functions of PARP, including SSBR, which results in the accumulation of SSBs. These SSBs convert to DSBs as a result of replication fork collapse, during DNA replication in the S phase of the cell cycle [35]. The predominant S-phase DSB repair pathway is HR, requiring function of the BRCA among other HR proteins [17,35]. In the case of HR-deficient tumours, the newly formed DSBs become lethal [35]. This is termed ‘synthetic lethality’ and is a key mechanism underlying the therapeutic success of PARPi [36]. Additionally, PARPi can induce PARP trapping on the DNA. This occurs as the result of an allosteric effect of the PARPi binding to the catalytic site, inducing a more potent bond between the DNA binding domain of the PARP enzyme and the nuclear DNA of the cell. The trapping effect results in the generation of more complex DNA lesions, which require the involvement of various DNA repair pathways (HR, Fanconi pathway, template switching, ATM/FEN1/Polβ), and are consequently considered more cytotoxic to tumour cells [35]. The ability to trap PARP1 varies between different inhibitors, with talazoparib having the strongest trapping ability, whilst veliparib has the weakest [35,37].

## 4. Current Clinical Applications of PARP Inhibitors in Cancer

PARP inhibitors (olaparib, niraparib, rucaparib and talazoparib) are currently approved as a monotherapy by the US Food and Drug Administration (FDA) and European Medicines Agency (EMA) for breast, ovarian, fallopian, primary peritoneal, prostate and pancreatic cancer. These approvals are all based on the ‘synthetic lethality’ principle, where only patients with a companion diagnostic (CDx) test result demonstrating a BRCA mutation (BRACAnalysis), HR mutation (FoundationOne CDx) or high genomic instability score (GIS) (MyChoice HRD CDx), as a marker of poor HR repair are deemed eligible for PARPi therapy [38,39,40,41,42,43,44,45]. Hence, the HR status and activity of HR genes are important determinants of therapeutic response to PARPi. There are numerous proteins involved in the HR repair pathway. Six key HR proteins (BRCA1, BRCA2, ATM, RAD51, MRE11, PALB2) have previously been demonstrated to be associated with the therapeutic response to PARPi [38,39,40,41,46,47]. Alterations of these HR proteins may render tumour cells more susceptible to PARPi monotherapy, due to the lack of HR repair.

In addition to its role as a monotherapy, PARPi may also prove useful in combination with other therapeutics. Immunotherapies and targeted therapies against oncogenic factors PI3K, MEK and CDK 4/6 are thought to reduce HR repair proficiency through various mechanisms. This would allow ‘synthetic lethality’ to occur with the addition of PARP inhibitors, rendering them promising treatment combinations [48]. Additionally, combining PARP inhibitors with DNA-damaging agents, such as chemotherapy and radiation therapy, could prevent repair of treatment-induced DNA damage. These approaches have been widely investigated in ovarian and breast cancers [48,49,50].

## 5. The Role of the DDR PARP Enzymes in GI Cancer Development and Progression

Currently, the prognosis for GI cancers remains poor while their incidence remains high [1,2]. Therefore, novel treatment approaches are needed to positively impact the prognoses of GI cancer patients. Increasing evidence supports a role for PARP enzymes in the development and progression of GI cancers, highlighting the potential for PARPi as a therapeutic strategy in these patients.

### 5.1. Oesophageal Cancer

The most common histological subtypes of oesophageal cancer are squamous cell carcinomas (OSCC) and adenocarcinomas (OAC). PARP1 is thought to aid cancer progression by promoting cell proliferation in OSCC. This has been demonstrated in vitro by a number of studies, with PARP inhibition/silencing resulting in decreased OSCC cell proliferation, due to increased accumulation of DNA damage and alteration of PARP1 function in the G2/M phase checkpoint pathway [51,52,53]. Furthermore, PARP2 mRNA expression was demonstrated to be significantly higher in oesophageal cancer tumour samples when compared to surrounding matched normal tissue. As PARP2 and PARP1 play similar roles in DDR, this may suggest an oncogenic role for both enzymes in oesophageal cancers [54]. Furthermore, PARP4 was identified as a marker of cancer stem cells (CSC) in OSCC, but not in OAC [55]. As CSCs are thought to initiate tumours and aid their progression through treatment resistance among other mechanisms, this may indicate an oncogenic role for PARP4 in OSCC [56].

### 5.2. Gastric Cancer

The *Helicobacter pylori* (*H. pylori*) bacterium is a leading global cancer-causing pathogen, primarily associated with gastric cancer [57]. Gastric cancer patients with *H. pylori* infection were demonstrated to express higher levels of PARP1 when compared to *H. pylori* negative gastric cancer patients [58,59]. *H. pylori* releases an unidentified factor that stimulates PARylation by the PARP1 enzyme [60]. The resultant overexpression and over-activity of PARP1 results in the switch of cellular preference to the NHEJ DSB repair pathway instead of HR, promoting genomic instability [61]. Furthermore, PARP1 was demonstrated to promote proliferation in gastric cancer cell lines, thought to be a result of FOXO3A-mediated cell cycle arrest suppression [62]. Hence, these PARP1-mediated effects are thought to aid tumour initiation in gastric cancer. This is supported by the increased PARP1 mRNA expression demonstrated in tumour samples, compared to surrounding matched normal tissue [59]. Furthermore, increasing PARP1 levels positively correlate with advanced gastric tumour stage, metastases and poorer survival rates, supporting a role for PARP1 in disease progression [58].

### 5.3. Hepatic Cancer

Hepatocellular carcinoma (HCC) has been demonstrated to have higher levels of PARP1 and PARP2 expression when compared to matched non-malignant tissue [63,64,65,66]. HCC tumours with increased PARP1/2 protein expression also exhibited a clinically poorer prognosis, with decreased overall and disease-free survival rates in addition to increased recurrence rates, tumour marker (alpha feto-protein) levels, invasion, metastases and tumour size, supporting a role for PARP1/2 in disease progression [64,66,67]. This is supported by a number of in vitro and in vivo studies, which have demonstrated pro-tumourigenic effects of PARP1 in HCC including increased proliferation, growth, angiogenesis, invasion and metastatic gene mRNA upregulation [64,68]. Supporting this, a number of in vitro studies in HCC have demonstrated that PARP inhibition results in an increase in apoptosis via the mitochondrial pathway [68,69]. However, Radnai et al., demonstrated an opposing effect with PARPi treatment inhibiting HCC apoptosis, an effect similarly observed in pre-malignant alcoholic liver disease [70,71]. This warrants further exploration of the role of PARP in HCC apoptosis.

One of the roles of PARP1 in the DDR is to recruit the ALC1 (activated in liver cancer 1) protein, amplified in over 50% of HCC tumours [25,72]. ALC1 is a chromatin modifier that promotes repair of DNA strand breaks, promotes proliferation, downregulates p53 expression and inhibits apoptosis. PARylation is thought to be the key regulator of ALC1; hence, PARP inhibition is hypothesized to significantly decrease tumourigenesis by preventing its upregulation [72,73]. Long non-coding RNAs (lncRNA) and microRNAs (miR) play a role in the upregulation of PARP1 and PARP2 levels in HCC, via *lncPARP1* or *lncPTTG3P* and *miR-149*, respectively [64,74,75]. This identified novel epigenetic targets that may achieve PARP1/2 inhibition in HCC.

### 5.4. Pancreatic Cancer

Pancreatic ductal adenocarcinomas (PDAC) comprise more than 85% of pancreatic cancers [76]. Acinar cell carcinomas, with a prevalence of less than 1%, are associated with a better overall prognosis than PDACs [76]. Additionally, they express PARP1 in almost 100% of tumour cells [77]. PARP depletion in acinar tumours in vivo was demonstrated to decrease proliferation, increase cell death and reduce acinar-to-ductal metaplasia (ADM) by driving clonal expansion of the prognostically favourable acinar malignant cells, suggesting PARPi as a promising therapeutic in early pancreatic cancer oncogenesis [77]. However, the application of clonal manipulation in the ADM cancer-initiating phase is limited by the clinical ability to detect patients in this sub-clinical pre-malignant phase.

PDACs have lower expression of PARP1; however, nuclear PARP expression has been demonstrated to positively correlate with an approximate three-fold benefit in overall survival in PDAC patients [77,78]. PDAC are unique within GI malignancies in that increased expression of PARP1 is related to a favourable prognosis. However, cytoplasmic PARP1, rather than nuclear, has been demonstrated to suppress the extrinsic pathway of apoptosis and promote survival, suggesting that subcellular localization of PARP1 may be important for patient stratification [79]. Nonetheless, PARP1 is more abundant in the nucleus, suggesting that the current clinical success of PARPi is heavily reliant on DNA repair inhibition and synthetic lethality, rather than the inhibition of cytoplasmic PARP1-mediated proliferation and survival, in pancreatic cancer [80].

### 5.5. Colorectal Cancer

PARP1 levels have been demonstrated to positively correlate with colorectal cancer (CRC) progression [81,82,83]. At early stages, PARP1 is thought to play an anti-tumour role in CRC by repairing DNA damage that would otherwise result in mutagenesis [83]. However, during disease progression, PARP1 activates NFκB, which promotes the secretion of pro-inflammatory cytokines (IL-1β, IL-6), allowing tumour-promoting inflammation. IL-6 also acts on the STAT3 pathway, ultimately resulting in cyclin D1 overexpression and cell proliferation [83,84]. Furthermore, NFκB activation results in the upregulation of metastasis-related proteins, such as integrins and matrix metalloproteinases (MMP) [85]. This PARP1-mediated progression is supported by evidence of higher PARP1 expression in tumours of patients with liver metastases than of their non-metastatic counterparts [86,87].

## 6. Homologous Recombination Status: A Predictive Biomarker of PARP Inhibitor Sensitivity in GI Cancers

The use of PARPi monotherapy in GI cancers is currently being investigated in clinical trials. Currently, the majority of approved indications for PARPi in all cancer types are based on the synthetic lethality principle, in which PARPi impedes SSB repair, ultimately leading to DSBs and cancer cell death due to HR deficiency [36]. Pancreatic cancer is the only GI cancer type with an EMA and FDA approved indication for a PARPi, olaparib, requiring the tumour to be BRCA-mutated per the BRACAnalysis CDx test, in line with the synthetic lethality principle [38,42]. This approval was granted based on the improved progression-free survival (PFS) achieved with olaparib in BRCA-mutated metastatic patients in the phase III POLO trial [88]. BRCA mutations were demonstrated to be present in up to 8% of pancreatic cancer patients, with a lesser prevalence of other HR mutations, such as in PALB2 and ATM [89,90,91,92]. The potential of extending the use of PARPi to patients with non-BRCA HR deficiencies is being investigated in several phase II trials [93,94,95]. One of these trials is investigating olaparib in pancreatic cancer patients with various HR gene alterations. Published preliminary results from this trial demonstrated PFS times ranging from 1.4–10.3 months, as median survival time has not yet been reached [93]. However, a randomized double-arm study is required to properly evaluate the efficacy of olaparib in HR-mutated pancreatic cancer patients.

Furthermore, synthetic lethality is being investigated in other GI cancer types. OSCC cell lines treated with a PARP inhibitor demonstrated an increased formation of RAD51 foci, suggesting increased HR repair [96]. Hence, the loss of this HR repair is hypothesized to result in an improved therapeutic response to PARP inhibition in OSCC. Moreover, OSCC sensitivity to PARP inhibition positively correlates with the amount of baseline DSBs, implicating a possible non-BRCA DSB repair defect not yet identified in some OSCCs [53,97]. While OSCC is in the pre-clinical phase, other GI cancers are currently undergoing clinical trials investigating PARPi with HR status as a biomarker. The concept of synthetic lethality is being investigated for the first time in OAC by a single-arm phase II trial of niraparib in OAC patients with an HR gene alteration or loss of heterozygosity (LOH). This trial further extends to include gastric cancer patients with an HR gene alteration or LOH (NCT03840967) [98]. This succeeds the results of preclinical studies demonstrating HR proteins, ATM and RAD51C as mediators of therapeutic response to olaparib in gastric cancer in vitro and in vivo mouse models [99,100]. Furthermore, olaparib is also being investigated in biliary cancer patients with an HR gene aberration by a single-arm phase II trial (NCT04042831) [101].

In CRC, ATM-depleted cell lines exhibited enhanced sensitivity to olaparib when compared to wild-type cell lines [102]. ATM mutations were demonstrated in up to 18% of CRC patients, who could potentially be ideal candidates for PARP inhibition therapy [103]. However, trials investigating ATM status as a potential biomarker for PARPi in CRC have not yet been conducted. Nonetheless, a phase II trial investigated olaparib in CRC patients with microsatellite instability (MSI) [104]. MSI status was used as a surrogate marker of MRE11 deficiency, due to a previously identified association between MSI tumours and MRE11 mutations [46,105]. The trial results did not demonstrate a significant difference in OS/PFS between the MSI patients and microsatellite stable (MSS) patients [104]. Hence, stratifying patients by MSI status was not an efficient approach, suggesting that MRE11 or other HR protein status could be more effective in identifying good responders to PARPi. Taken together, these data suggest that HR status is potentially a strong predictor of PARPi response in GI cancers and could be used to identify patients who would potentially benefit from PARPi.

## 7. The Role of DDR PARP Enzymes in the Response of GI Cancer to Radiation and Chemotherapy

The use of PARPi monotherapy in GI cancers is currently being investigated in clinical trials. In addition to the role of PARP in tumour development and progression of GI cancers, the DDR PARP enzymes play a role in treatment response. Their ability to repair chemoradiotherapy (CRT)-mediated DNA damage could confer resistance to these agents. This highlights a potential role for PARPi in enhancing the efficacy of cytotoxic DNA-damaging agents. This targeting approach is currently undergoing pre-clinical and clinical investigation in GI cancers.

### 7.1. Pre-Clinical Evidence

#### 7.1.1. Radiotherapy

Ionizing radiation (IR) induces DNA strand breaks. Whilst SSBs are more abundant, it is DSBs that are predominantly lethal to the cell. PARP inhibitors are thought to sensitize GI cancer cells to IR by two mechanisms: (i) inhibition of SSB repair resulting in the accumulation of DSBs and (ii) inhibition of DSB repair. This was illustrated in oesophageal, pancreatic, hepatic, gallbladder and CRC cancers where combination therapy (PARPi/IR) in cell lines resulted in increased DSBs and reduced tumour cell growth and survival, compared to IR alone [47,106,107,108,109]. Levels of γH2AX, a marker of DSBs, classically decline 24 h after IR as a result of DNA repair. However, PARP inhibition resulted in the persistence of γH2AX foci in OSCC, CRC and pancreatic cancer cell lines, suggesting inhibition of DSB repair [47,107,110]. Moreover, it has been previously demonstrated that the loss of SSB repair alone is not enough to radio-sensitize tumour cells, further supporting an additional role of PARP inhibition in loss of efficient DSB repair [111]. Similar radio-sensitization was demonstrated when PARP inhibition was combined with high linear energy transfer (LET) radiation [112,113].

Xenograft models revealed enhanced tumour growth delay in OSCC and CRC with increased median survival associated with the addition of PARP inhibition to IR, compared to IR alone [47,106,110,114,115]. Moreover, in CRC xenografts, PARP inhibition was demonstrated to increase the tumour growth delay effect of CRT (radiotherapy with either irinotecan, oxaliplatin, or 5-Fluorouracil) [110]. Enhanced tumour growth inhibition was also demonstrated with combined PARP2 silencing and IR treatment in HCC xenografts, when compared to IR alone [66]. No significant tumour growth delay was demonstrated in BRCA mutant or wild-type pancreatic xenografts, with other GI cancer sites yet to be studied [108].

Hypoxia is associated with radiotherapy resistance due to the lack of DNA damage fixation by oxygen, resulting in the repair of IR-induced DNA damage in hypoxic cells [116]. However, despite the ability to repair the IR-induced damage, hypoxic cells have been demonstrated to downregulate DSB repair proteins such as RAD51 and BRCA1 [47,111]. This is significant as RAD51 has been demonstrated to be associated with the repair of PARP-induced lesions, resulting in PARPi resistance [96,117]. Combination treatment of olaparib and IR significantly increased apoptosis in hypoxic cells, when compared to normoxic OSCC in vitro [47]. This effect was seen in other cancer cell lines: gliomas, non-small cell lung cancer and prostate cancer [118,119]. Hence, this may suggest that PARPi could have a potent radio-sensitizing effect in radio-resistant hypoxic tumour cells by exploiting their reduced DSB repair ability [47].

These results suggest that PARPi may improve radioresponse in GI cancers. Currently, combination therapy of PARPi with IR in OAC and gastric cancer has not been explored. However, we have previously demonstrated that *PARP1* mRNA is increased in pre-treatment OAC tumour samples from patients who exhibited subsequent poor pathological response to CRT, implicating a potential role for PARP1 in the resistance of OAC to CRT [120].

#### 7.1.2. Chemotherapy

Chemotherapeutic agents can induce a plethora of DNA lesions, which are repaired by a variety of mechanisms. As the DDR PARP enzymes play multiple roles in DNA repair pathways (BER, NER, SSBR, DSB repair), as reviewed by Chaudhuri et al., PARPi are hypothesized to hinder repair of chemotherapy-induced DNA damage and improve therapeutic response to these agents [25].

Alkylating agents result in alkylation of the DNA bases, which can ultimately result in SSBs, requiring PARP-mediated BER and SSB repair [25,121,122]. Hence, inhibition of this PARP1-mediated DNA repair may allow the damage to persist, resulting in strand breaks and ultimately tumour cell death [121]. This is supported by in vitro studies of temozolomide (TMZ) and PARPi combination therapy, which results in an increase and persistence of γH2AX with reduced colony growth of HCC, CRC and OSCC cell lines, when compared to TMZ therapy alone [114,123,124,125,126]. Furthermore, the use of TMZ with PARPi in CRC mouse xenografts resulted in significant tumour growth delay, when compared to TMZ alone. Unfortunately, the combination was associated with significant weight loss in the mice, up to 20% of baseline, requiring dose modifications, and warrants further safety investigation [114].

DNA damage induced by platinum-based therapy is largely repaired by the NER pathway, in which PARP1 facilitates the NER proteins’ access to the DNA lesions through chromatin modification [25,127,128]. Therefore, the loss or inhibition of PARP in combination with cisplatin demonstrates greater growth suppression of HCC and OSCC cell lines, reduced cell viability in OAC and a synergistic effect in CRC cell lines, when compared to the cisplatin-only control groups [96,123,129,130,131]. Furthermore, an increase in DSB formation in OSCC was demonstrated by the significantly higher levels and persistence of γH2AX foci in the combination treatment group, compared to cisplatin alone. This suggests a beneficial effect of PARPi combination with cisplatin in GI cancers. However, this effect was also demonstrated with the addition of 5-flourouracil (5-FU) to cisplatin in OSCC, with no statistically significant difference between the two combinations (PARPi/cisplatin vs. 5-FU/cisplatin) [123]. Hence, the effect of PARPi could potentially be additive, where it increases the DNA damage load, similar to 5-FU, rather than a sensitizing effect where it specifically inhibits the repair of platinum-induced damage. Further investigation is warranted to ascertain the role of PARPi use in combination with platinum-based therapy and a potential benefit over combination chemotherapy (5-FU/cisplatin) use in GI cancer patients.

Topoisomerase-1 (Top1) inhibitors result in formation of SSBs, which require PARP1-mediated SSB repair to prevent DSB formation and tumour cell death [132,133]. Irinotecan, or its active metabolite SN-38, are common Top1 inhibitors administered to GI cancer patients [134]. Their combination with PARPi in vitro increased γ-H2Ax foci and enhanced colony growth suppression in OSCC, CRC and gastric cancer cell lines [123,135,136,137,138,139]. Moreover, xenograft studies revealed significantly reduced tumour growth and tumour growth delay in gastric and CRC models with the combination treatment, with only one study demonstrating unacceptably significant mouse weight loss [114,135,138,139,140,141,142]. Interestingly, gastric cancer in vivo models with high ATM expression exhibited more resistance to irinotecan monotherapy but achieved a more marked improvement in tumour growth inhibition when combined with PARPi therapy [139]. This highlights the role of HR status as a predictive biomarker of PARPi combination therapy response.

Antimetabolite agents induce a wide range of DNA damage, which is repaired by different pathways. Gemcitabine-induced lesions are thought to be removed by NER, hence a potential target for PARP inhibition [143]. The combination of PARP inhibition with gemcitabine demonstrated reduced tumour cell viability with an increase in apoptotic cells and caspase activity in two pancreatic cancer cell lines. This was further supported in xenograft mouse models, where increased apoptosis, decreased tumour weight and increased overall survival were demonstrated with combined treatment, when compared to gemcitabine alone [144]. The incorporation of 5-FU into DNA is predominantly repaired by the pyrimidine sub-pathway of BER or mismatch repair (MMR) in which the DDR PARP enzymes are not thought to play a role [25,122,145]. This translated to pre-clinical studies where PARP inhibitors demonstrated no significant sensitization to 5-FU in OSCC and CRC [96,110,141,146].

The primary mechanism of anti-microtubule agents is to halt tumour cell mitosis by disrupting microtubule function [147,148]. As they do not directly target DNA, their combination with PARPi is not well-studied. The use of a fluzoparib-paclitaxel-apatinib (a VEGFR-2 inhibitor) three-drug combination in gastric cancer patient-derived xenografts resulted in a 75.6% growth inhibition, compared to control. However, the two-drug combination fluzoparib-apatininb resulted in a 72.8% growth inhibition, suggesting that the effect is mostly due to the PARPi/VEGFR-2 inhibitor combination [149].

### 7.2. Clinical Trials Assessing PARPi and Cytotoxic Therapy Combination Treatment Approaches

Current evidence from pre-clinical studies suggests that combining PARPi with radiotherapy and chemotherapeutic agents used in the treatment of GI cancers may improve treatment response. A number of clinical trials are currently evaluating the safety and efficacy of this approach (Table 1).

#### 7.2.1. Oesophageal Cancer

As pre-clinical success was achieved with PARPi/platinum analogue combinations in OSCC, two oesophageal cancer patients were enrolled in a phase I clinical trial of this combination therapy [96,123,150]. The trial examined the toxicity profile and efficacy of a commonly used regimen for oesophageal cancer, carboplatin/paclitaxel with veliparib [150,151]. Due to high grade neutropenia, the maximum tolerated dose was declared as 100 mg in combination with carboplatin/paclitaxel, one-third of what is currently used for monotherapy in clinical practice. In terms of response, both patients had stable disease with a 0% objective response rate (ORR), defined as the proportion of patients with reduction in tumour burden of a predefined amount [150]. A phase I trial investigating fluzoparib with paclitaxel-apatinib in gastroesophageal adenocarcinoma published preliminary results suggesting that the combination is safe to use and the median PFS is 4.9 months. However, the benefit of this regimen remains unknown, warranting further investigation [152]. Another combination currently being investigated in OAC is the combination of FOLFIRI (5-FU, irinotecan, folinic acid) with rucaparib (NCT03337087) [153].

#### 7.2.2. Gastric Cancer

Paclitaxel is administered as second-line treatment for relapsed gastric cancer patients [154]. While its combination with fluzoparib is still in early phase trials, its combination with olaparib is at advanced clinical trial stages [152]. A double-blind randomized phase II trial demonstrated a significant overall survival (OS) benefit with combination olaparib-paclitaxel therapy, compared to placebo/paclitaxel, but no significant PFS or ORR benefit [155]. Interestingly, this OS benefit was greater in patients with low ATM protein expression [155]. It was previously demonstrated that in ATM-deficient gastric xenografts, veliparib/irinotecan did not result in a superior tumour growth inhibition compared to veliparib alone, suggesting that the benefit could be attributed to veliparib alone and synthetic lethality [139]. Hence, it would be interesting to compare OS in patients receiving olaparib/paclitaxel versus olaparib alone in low ATM expressing gastric cancer patients.

Nonetheless, the OS benefit observed with olaparib/paclitaxel led to the GOLD trial, a phase III double-blind randomized trial with the same combination. Unfortunately, it did not demonstrate a significant benefit in OS or PFS in preliminary results [156]. Nonetheless, given the positive results of PARPi with irinotecan in vivo, a phase I study was performed in 20 gastric patients receiving veliparib with first-line treatment option FOLFIRI combination therapy [154]. The combination was deemed safe to evaluate in a phase II setting. ORR was 15% for gastric patients in this trial, suggesting a potential benefit requiring validation in a larger cohort [157].

#### 7.2.3. Hepatic and Biliary Cancer

TMZ in combination with veliparib has demonstrated promising results in vitro [126]. The combination of TMZ with veliparib was demonstrated to be safe with no pharmacokinetic interactions in a phase I study of solid malignancies, including HCC [158]. The safety of this treatment combination was also demonstrated in a single arm phase II trial in first-line refractory HCC patients, however, none of the participants had a radiological response. The majority of patients had progressive disease whilst a minority had stable disease [159]. No strong evidence exists for the efficacy of any cytotoxic agent use in HCC [160]. Hence, it would be worthwhile to investigate PARPi in combination with other therapeutics for this cancer type. Pre-clinical studies have investigated combination of PARPi with arsenic trioxide, histone deacetylase inhibitors and NFκB inhibitors in HCC [161,162,163]. The combination of PARPi with DNA-damaging agents in biliary cancer has not been investigated in clinical trials before, but currently a phase I/II trial is evaluating the combination of rucaparib with FOLFIRI (5-FU, irinotecan, folinic acid) in this cancer type (NCT03337087) [153].

#### 7.2.4. Pancreatic Cancer

The most commonly used first-line chemotherapeutics in pancreatic cancer are FOLFIRINOX (5-FU, irinotecan, oxaliplatin, folinic acid) and gemcitabine [164]. A phase I study of gemcitabine with olaparib in solid tumours demonstrated acceptable safety with an ORR of 10%, the majority of responders being pancreatic cancer patients [165]. These results correlate with the pre-clinical investigation of this combination in pancreatic cancer [144]. The combination of veliparib with gemcitabine-cisplatin is currently undergoing a phase II trial, in BRCA/PALB2-mutated patients (NCT01585805) [166]. Furthermore, veliparib was investigated in combination with CRT (gemcitabine and IR) in locally advanced patients in the VelGemRad phase I trial. The trial demonstrated an acceptable safety profile, with promising efficacy results of a 15-month median OS and only one patient (out of 30) exhibiting progressive radiological disease [167].

A Phase I trial assessing the combination of olaparib with irinotecan, cisplatin and mitomycin C achieved a promising ORR of 23%. Unfortunately, this trial demonstrated an unacceptable safety profile for this treatment [168]. Nonetheless, when veliparib was combined with FOLFIRI, a favourable safety profile was achieved in addition to a 14% ORR [157]. As a result, a randomized phase II trial was executed to observe the effect of adding veliparib to second-line FOLFIRI. Preliminary data shows increased toxicity with no OS or PFS benefit. Data analysis based on BRCA and DDR status is pending [169]. Additionally, fluzoparib is undergoing investigation with FOLFIRINOX in two trials, based on disease stage (NCT03026881, NCT04425876).

A phase I/II trial of veliparib with FOLFOX (5-FU, oxaliplatin, folinic acid) has demonstrated an acceptable safety profile. The primary endpoint ORR at 26% was met, while the ORR was even higher at 30–58% for patients with a family history suggestive of breast and ovarian cancer syndrome or DDR mutation [170]. This warrants further investigation with a randomized double-arm clinical trial to validate a benefit of adding veliparib to FOLFOX.

#### 7.2.5. Colorectal Cancer

FOLFIRI is a commonly used treatment regimen in CRC [171]. Various pre-clinical studies have demonstrated the benefit of combining irinotecan with PARPi in CRC. A Phase I trial of FOLFIRI with veliparib demonstrated an acceptable safety profile with an ORR of 22% [157]. However, a randomized phase II investigation of this combination yielded no OS, PFS or response rate benefit with the addition of veliparib, when compared to the standard of care [172]. Nonetheless, the combination of FOLFIRI (5-FU, irinotecan, folinic acid) with rucaparib is currently being investigated in CRC, amongst other GI cancers (NCT03337087) [153].

Although TMZ is not routinely used in the treatment of CRC, its combination with veliparib has been studied in a single arm phase II trial [171,173]. The trial achieved the required disease control rate of its primary endpoint, with an acceptable safety profile [173]. Currently, TMZ in combination with olaparib is being investigated in a double-armed non-randomized phase II trial, with results expected to be published in 2023 (NCT04166435) [174].

PARPi improved the anti-tumour effect of concurrent radiotherapy with 5-FU, among other agents, in CRC xenografts [110]. This is relevant in the context of rectal cancer, where CRT is the standard of care for locally advanced disease [175]. A phase I study demonstrated an acceptable safety profile of adding veliparib to an IR and capecitabine (5-FU prodrug) CRT regimen in rectal cancer [176]. A pCR rate of 29% was achieved, comparatively higher to the current 16% pCR average for rectal tumours [8,176]. In a Phase II trial of this combination, preliminary results did not show a benefit over the standard of care in achieving pCR or improvement in the primary surrogate endpoint, the neoadjuvant rectal (NAR) score [177].

Noticeably, despite the promising results from pre-clinical evaluation of PARPi and IR combination treatment, there is a marked absence of clinical trials investigating an IR and PARPi combination. This is particularly important given that approximately 55–80% of patients with oesophageal, gastric, pancreatic or rectal tumours receive radiotherapy [178]. Hence, further clinical investigation of PARPi combined with radiation therapy in GI cancers is warranted.

Nonetheless, preclinical evaluation of PARPi combined with DNA-damaging agents has demonstrated encouraging results in achieving improved therapeutic responses. Clinical studies have also demonstrated promising results in early phase trials, with more success required in the later phase trials. There are a number of current clinical trials investigating the combination of PARPi and DNA damaging agents in GI cancers (Table 1), which will identify if this novel treatment strategy may be beneficial for these patients.

## 8. The Landscape of PARP and HR in Gastrointestinal Tumours

**Figure 2:**
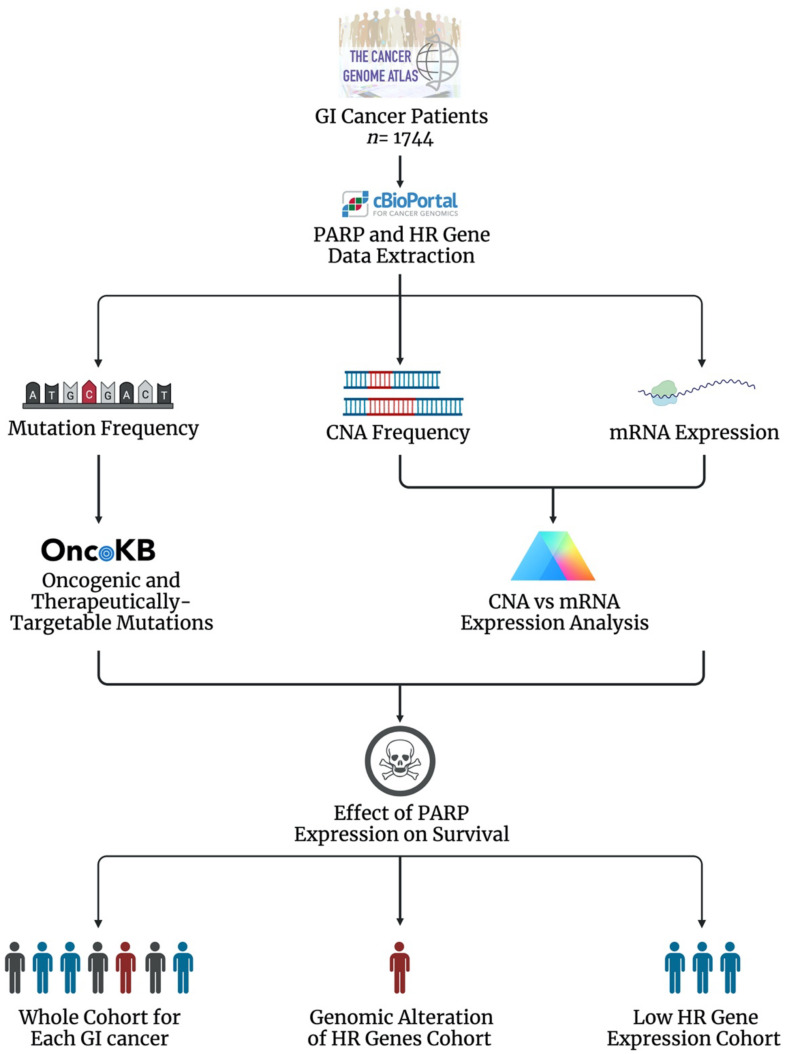
Workflow diagram of data collection and analysis [32,179,180,181,182,183,184,185,186,187].

The current literature suggests that the DDR PARP enzymes play a role in the development, progression and treatment response of GI cancers, supporting PARPi as a therapeutic approach. Evidence also supports HR status as a predictor of response to PARPi. However, the global genomic and transcriptomic landscape of the PARP genes and associated HR genes in GI cancers is largely unknown. To investigate this, we profiled the genomic and transcriptomic landscape of the DDR PARP genes (*PARP1*, *PARP2*, *PARP3* and *PARP4*) and six key HR genes (*BRCA1*, *BRCA2*, *ATM*, *RAD51*, *MRE11* and *PALB2*) across 1744 gastrointestinal cancer patients (87 oesophageal adenocarcinoma (OAC), 95 oesophageal squamous cell carcinoma (OSCC), 440 gastric adenocarcinoma (GAC), 369 hepatocellular carcinoma (HCC), 36 cholangiocarcinoma (CCA), 184 pancreatic adenocarcinoma (PAAD), 378 colon adenocarcinoma (COAD) and 155 rectal adenocarcinoma patients (READ)) using publicly available datasets (The Cancer Genome Atlas (TCGA) consortium) (Figure 2) [179,180,181,182,183]. The oncogenic potential of mutations was assessed using the Oncology Knowledge Base (OncoKB), which curates information on the effects and therapeutic implications of specific gene alterations (Figure 2) [184]. mRNA expression was presented as RSEM ± the standard error of the mean (SEM), as transcript quantification was obtained from the RNA-sequencing data using the RNA-Seq by Expectation Maximization (RSEM) program [185]. The full method is available in Supplementary Materials.

### 8.1. The Genomic and Transcriptomic Landscape of the DDR PARP Genes in GI Cancers

The frequency of DDR PARP gene alterations (mutation or copy number alteration (CNA)) was investigated in GI cancers. Overall, PARP mutations were of a low frequency, with a mean frequency of 1%, across the GI cancer cohort assessed (Table 2). The majority of *PARP1* mutations identified in the OncoKB knowledge base were demonstrated to result in a loss-of-function [184]. The frequency of PARP CNAs was also low, with a mean frequency of 1.6% (Table 2). The majority of these CNAs were amplifications (82%). Interestingly, we demonstrated a variation in CNA frequency between the oesophageal cancer subtypes. *PARP1* and *PARP2* CNA frequencies were higher in OSCC, while *PARP3* and *PARP4* CNA frequencies were higher in OAC (Table 2). Variations in the genes affected by CNA between the oesophageal histological subtypes were previously demonstrated across a variety of genes, with only a minority of CNAs demonstrated in the same gene in both subtypes [188]. This may suggest DDR PARP CNAs as a potentially distinguishing molecular feature between the oesophageal subtypes; however, further studies are needed to validate this as a potential diagnostic marker and identify the functional significance of this variation.

**Figure 3:**
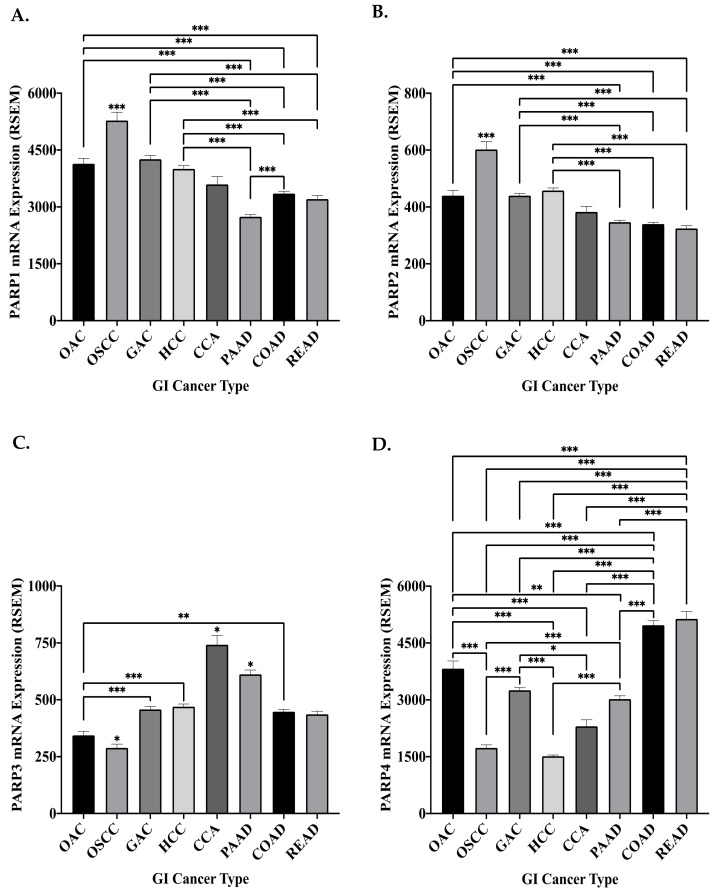
Mean mRNA expression of the DNA damage response PARP genes in GI cancers. The mean mRNA expression of (**A**) *PARP1* (**B**) *PARP2* (**C**) *PARP3* and (**D**) *PARP4* in GI cancers. OAC, *n* = 97; OSCC, *n* = 94; GAC, *n* = 412; HCC *n* = 363; CCA, *n* = 36; PAAD, *n* = 177; COAD, *n* = 377; READ, *n* = 154. Data are presented as the mean RSEM ± SEM. Statistical analysis was performed using one-way ANOVA with Tukey’s multiple comparison post-hoc test, * *p* ≤ 0.05, ** *p* ≤ 0.01, *** *p* ≤ 0.001. When significance is not directly comparing two groups, it is significant in comparison to all other groups.

The mRNA expression of the four DDR PARP genes significantly varied across the GI cancers, with no distinguishable pattern (Figure 3). Interestingly, the mRNA expression of the DDR PARP genes was significantly altered between the oesophageal cancer subtypes (Figure 3). OSCC demonstrated significantly increased mRNA expression of *PARP1* (*p* < 0.0001) and *PARP2* (*p* < 0.0001), when compared to OAC patients. This is supported by a previous study, which demonstrated that PARP1 protein expression is significantly higher in OSCC, when compared to OAC in rat models [189]. Interestingly, in OSCC patients with *PARP1* chromosomal amplifications, the *PARP1* mRNA expression (mean RSEM 9826 ± 1050 SEM) was significantly (*p* = 0.0009) higher, when compared to *PARP1* mRNA expression (mean RSEM 4417 ± 270.8 SEM) in diploid (no CNA) OSCC patients. Similarly, this significant alteration (*p* = 0.0004) was demonstrated with *PARP2* (mean mRNA expression RSEM 1085 ± 208.7 SEM in patients with *PARP2* amplifications, when compared to RSEM 531 ± 21.2 SEM in diploid patients). This suggests chromosomal amplifications as a potential underlying mechanism of the altered PARP mRNA expression observed between the oesophageal cancer subtypes. As PARP1 was previously demonstrated to be associated with enhanced proliferation and enhanced DNA repair of cytotoxic agent-induced damage in OSCC, this suggests that the amplification-mediated increase in *PARP1* and *PARP2* expression in OSCC could potentially play a role in the development and progression of these tumours [51,190].

Contrastingly, a significantly higher mRNA expression of *PARP3* (*p* < 0.05) and *PARP4* (*p* < 0.0001) was demonstrated in OAC, when compared to OSCC. PARP3 is thought to facilitate the repair of DSBs, specifically in guiding the repair pathway choice (HR or NHEJ) [28,29]. OAC has been demonstrated to overexpress HR genes, when compared to normal matched tissue [191]. This may suggest that PARP3 overexpression is a potential driver of increased HR pathway selection in OAC, as PARP3 depletion has been previously demonstrated to reduce cellular HR efficiency [28]. HR repair is the major player in preventing genomic instability and apoptosis arising from unrepaired DSBs following endogenous or exogenous (CRT-induced) insult, permitting tumour survival and consequently progression [17]. Therefore, the increased expression of *PARP3* mRNA in OAC may have implications for cytotoxic therapy response in these patients and may suggest that inhibition of PARP3 could boost efficacy of treatment. However, assessing protein expression of DDR PARP genes in oesophageal cancer is required to fully ascertain their role in the development and progression of oesophageal cancers. In addition, investigation of the subcellular localization of PARP in oesophageal cancers should be investigated due to the impact on prognosis demonstrated in previous studies [77,78,79].

### 8.2. The Genomic and Transcriptomic Landscape of Key HR Genes in GI Cancers

The overall mutation frequency of the six HR genes in GI cancers was low, with a mean frequency of 2.3% (Table 3). A third (139/393 mutations) of the identified HR mutations in the GI cancer cohort are currently being therapeutically exploited by PARPi in other cancer types (breast, ovarian, prostate, pancreatic) in the clinic, as per OncoKB [184]. This suggests that these mutations could potentially be targeted in GI cancer patients by PARPi.

The CNA frequency of HR genes in GI cancers was low, with a mean frequency of 1%, and predominantly consisted of amplifications (66% of CNAs). Interestingly, *BRCA1* and *BRCA2* demonstrated the highest frequency of CNA across the GI cancer cohorts. HR amplifications, specifically *BRCA2* and *MRE11*, have been demonstrated to enhance PARPi resistance in pancreatic cancer cell lines and a single breast cancer patient, respectively [192,193]. This is thought to be the result of increased HR expression and proficiency, due to the gene amplifications, hence opposing synthetic lethality and resulting in a poorer therapeutic response. Although no substantial evidence exists for the role of HR amplifications in PARPi response, *BRCA2* being the most commonly amplified HR gene in GI cancers could prove to be an obstacle for PARPi. However, the prevalence of HR gene CNAs in the GI cancer cohort was low, which may suggest that CNA-mediated mechanisms of PARPi resistance are unlikely in this cohort.

**Figure 4:**
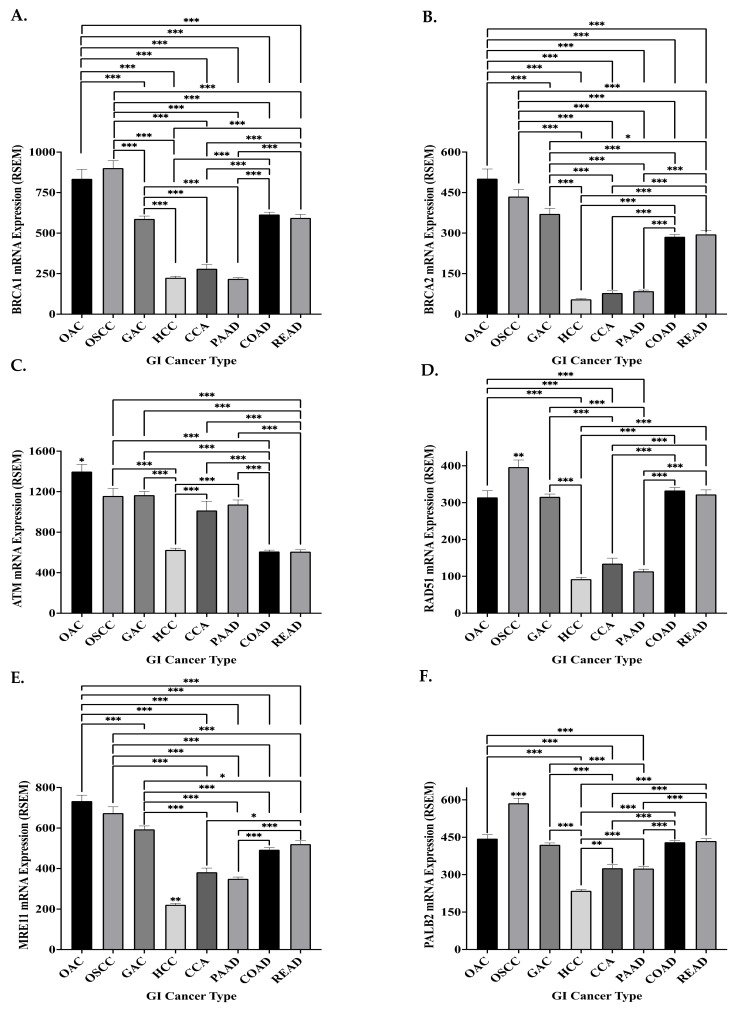
Mean mRNA expression of HR genes in GI cancers. Mean mRNA expression of (**A**) *BRCA1* (**B**) *BRCA2* (**C**) *ATM* (**D**) *RAD51* (**E**) *MRE11* and (**F**) *PALB2* in GI cancers presented as the mean RSEM ± SEM. OAC, *n* = 97; OSCC, *n* = 94; GAC, *n* = 412; HCC *n* = 363; CCA, *n* = 36; PAAD, *n* = 177; COAD, *n* = 377; READ, *n* = 154. Statistical analysis was performed using one-way ANOVA with Tukey’s multiple comparison post-hoc test, * *p* ≤ 0.05, ** *p* ≤ 0.01, *** *p* ≤ 0.001. When significance is not directly comparing two groups, it is significant in comparison to all other group.

The mRNA expression of the six key HR genes was highest in the oesophageal cancers (*p* < 0.05) and lowest (*p* < 0.05) (excluding *ATM* and *MRE11* in CCA) in HCC, CCA and PAAD patients (Figure 4). In agreement with the ‘synthetic lethality’ principle, low expression of HR genes has been demonstrated to be associated with improved response to PARPi in GI cancers, both in pre-clinical and clinical studies [46,100,155]. Hence, the low expression of HR genes in PAAD may explain the good response of PAAD patients to PARPi in clinical trials [194]. The data may suggest that the other GI cancers, especially the oesophageal cancers, could be less sensitive to PARP inhibition (Figure 4). However, mRNA expression alone is not a sufficient measure of protein expression and function. The mRNA expression of *ATM* was previously demonstrated to be reduced in HCC tissue compared to surrounding matched tissue, while ATM and RAD51 protein expression was demonstrated to be conversely increased in HCC tissue compared to surrounding tissue [195,196]. Current literature supports our findings in OAC, as mRNA overexpression of HR genes was previously demonstrated in this cancer type [191]. Interestingly, *RAD51* mRNA expression is significantly higher in OSCC, when compared to OAC patients (Figure 4). As RAD51 has been demonstrated to be associated with PARPi resistance, this suggests that OAC patients could potentially be more sensitive to PARPi [96,117].

However, discrepancies exist between this mRNA data and previously reported protein expression of the HR genes in pancreatic cancer, where BRCA1 protein expression was low in PAAD patients but RAD51 protein was overexpressed [197,198]. Additionally, post-transcriptional regulation of HR proteins has been previously demonstrated in GI cancers, specifically OAC [199]. Therefore, HR protein expression data is needed to fully determine the potential of HR expression to predict the sensitivity of GI cancers to PARPi. However, the mRNA expression data presented here may suggest that synthetic lethality is not an ideal approach for GI cancers, highlighting the need for novel PARPi and cytotoxic therapy combination targeting strategies.

### 8.3. The Prognostic Effect of PARP mRNA Expression in GI Cancers

The impact of PARP mRNA expression on overall survival (OS) in the GI cancer cohort was then investigated. The median PARP mRNA expression was used to stratify patients into high (expression values higher than the median) and low (expression values lower than the median) expression groups.

**Figure 5:**
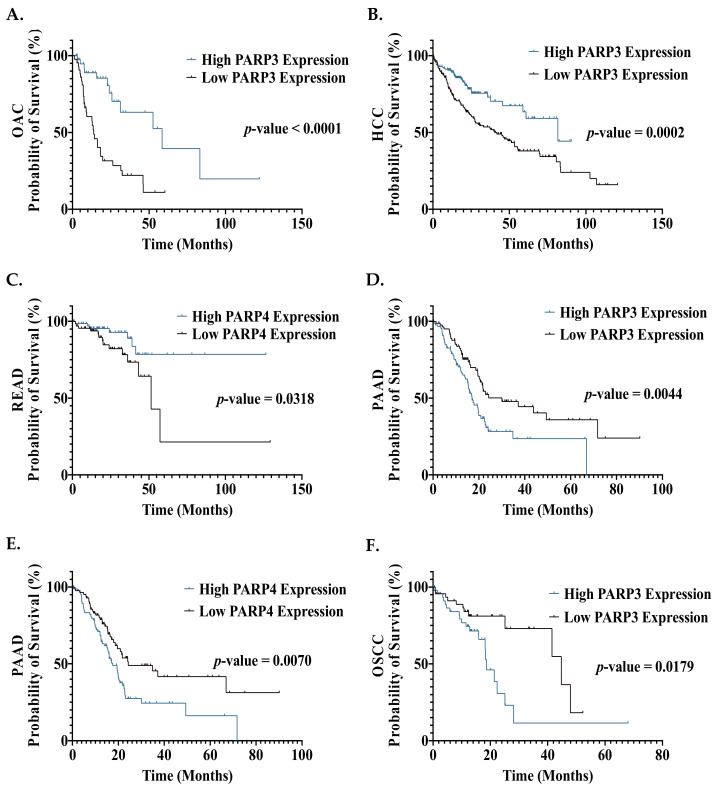
Effect of DDR PARP gene expression on overall survival outcomes of GI cancer patients. (**A**) OAC patients with high *PARP3* expression (*n* = 43) have significantly improved OS, when compared to OAC patients with low *PARP3* expression (*n* = 43) (median OS of 58.6 months vs. 13.9 months). (**B**) HCC patients with high *PARP3* expression (*n* = 181) have significantly improved OS, when compared to HCC patients with low *PARP3* expression (*n* = 181) (median OS of 81.7 months vs. 40.4 months). (**C**) READ patients with high *PARP4* expression (*n* = 76) have significantly improved OS, when compared to READ patients with low *PARP4* expression (*n* = 76) (median OS undefined vs. 51.5 months). (**D**) PAAD patients with high *PARP3* expression (*n* = 88) have significantly poorer OS, when compared to PAAD patients with low *PARP3* expression (*n* = 88) (median OS of 17 vs. 30 months). (**E**) PAAD patients with high *PARP4* expression (*n* = 88) have significantly poorer OS, when compared to PAAD patients with low *PARP4* expression (*n* = 88) (median OS of 17.5 months versus 24.3 months). (**F**) OSCC patients with high *PARP3* expression (*n* = 47) have significantly poorer OS, when compared to OSCC patients with low *PARP3* expression (*n* = 47) (median OS of 18.6 months versus 44.7 months). Statistical analysis was performed using a Kaplan–Meier survival analysis and log-rank (Mantel–Cox) test.

In the OAC and HCC cohort, patients in the high *PARP3* expression group demonstrated a significantly improved OS, compared to the low expression group (Figure 5A,B). Due to their role in HR promotion, high PARP3 levels are expected to result in enhanced repair of CRT-induced damage, leading to poor therapeutic responses, tumour progression and a poorer prognosis [28,29]. However, our findings demonstrated the opposite effect, suggesting a protective role for PARP3. As HR loss has been demonstrated to result in mutagenesis, PARP3-directed enhanced HR repair could potentially halt tumour progression by promoting genomic stability [200]. However, no current evidence demonstrates an improved prognosis of GI cancer patients with increased HR efficiency, warranting further investigation into the involvement of PARP3 in HR and alternative repair pathways. Furthermore, previous studies suggest that high PARP1 expression is associated with poorer survival outcomes in HCC and radioresistance in OAC [64,120]. Hence, this raises the need for enzyme-specific PARPi, as PARP1 could potentially be targeted in OAC and HCC while PARP3 would be spared. A major obstacle to this selective targeting approach would be the structural homogeneity of PARP 1-4 enzymes [24,31].

High *PARP4* expression was associated with a significantly improved OS in READ patients (Figure 5C); however, as the role of PARP4 in the DDR is currently unknown, the clinical utility of this is unclear.

High *PARP3* and *PARP4* expression was associated with a significantly poorer OS in the OSCC and PAAD cohorts (Figure 5D–F). This is not surprising for the PAAD cohort, given the clinical success of PARP inhibitors in this patient cohort [88]. These results are supported by a single-arm clinical trial by Tuli et al., assessing the PARPi veliparib in combination with CRT in pancreatic cancer patients, which demonstrated that patients with pre-treatment samples demonstrating high *PARP3* mRNA expression exhibited an improved OS in the trial [167]. This suggests a possibly enhanced response to CRT achieved with veliparib in patients with high PARP3 expression. However, as PARP3 inhibitory activity is weak with veliparib, further studies are needed to validate this effect [33,34]. The current success of targeting PARP1 in PAAD is thought to be exclusively related to the synthetic lethality principle and not the prognostic impact of PARP1 in PAAD. However, our findings suggest that PARP3/4 inhibition may achieve a therapeutic effect, without the need for synthetic lethality. Hence, the development of PARPi with potent anti-PARP3/4 activity is warranted.

Whilst only *PARP3* expression was demonstrated to negatively affect prognosis in this OSCC cohort (Figure 5F), previous studies have demonstrated that high PARP1 and PARP4 protein expression was associated with a significantly poorer OS in OSCC patients [51,55]. This study assessed mRNA expression, not protein expression, which may explain these differences. However, taken together, this may support a role for DDR PARP enzymes in the progression of OSCC, highlighting a potential therapeutic benefit from PARPi in these patients.

**Figure 6:**
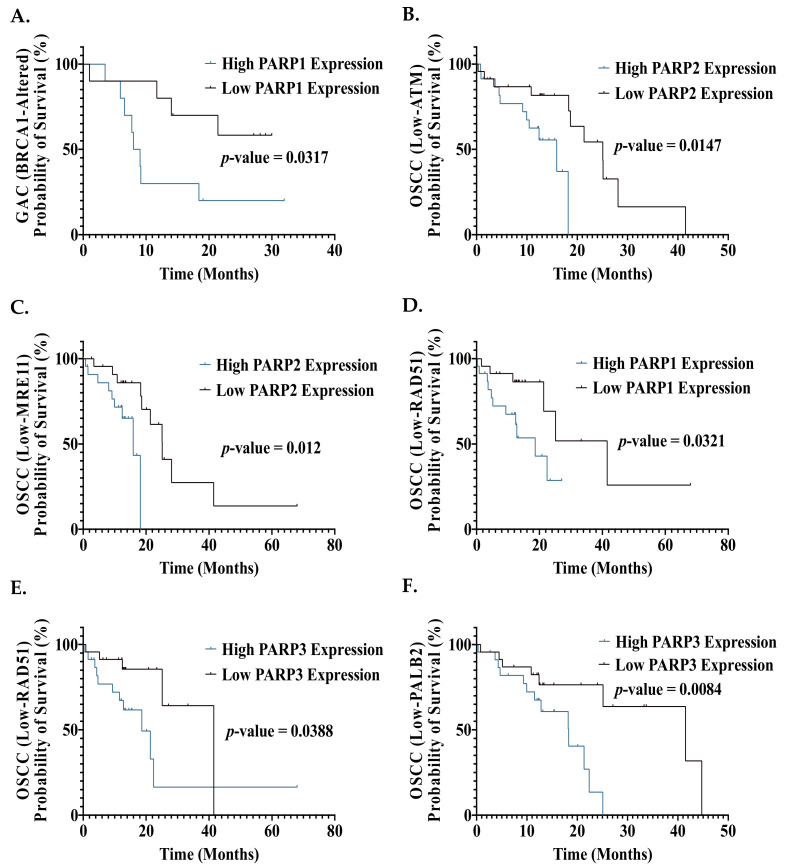
Effect of DDR PARP gene expression on overall survival outcomes of GI cancer patients with altered HR genes or low HR gene expression. (**A**) BRCA1-altered GAC patients with high *PARP1* expression (*n* = 11) have a significantly poorer OS, when compared to BRCA1-altered GAC patients with low *PARP1* expression (*n* = 11) (median OS of 8.5 months vs. undefined). (**B**) OSCC patients with low *ATM* and high *PARP2* expression (*n* = 23) have significantly poorer OS, when compared to OSCC patients with low *ATM* and low *PARP2* expression (*n* = 23) (median OS of 15.9 vs. 25.1 months). (**C**) OSCC patients with low *MRE11* and high *PARP2* expression (*n* = 23) have significantly poorer OS, when compared to OSCC patients with low *MRE11* and low *PARP2* expression (*n* = 23) (median OS of 15.9 vs. 25.1 months). (**D**) OSCC patients with low *RAD51* and high *PARP1* expression (*n* = 23) have significantly poorer OS, when compared to OSCC patients with low *RAD51* and low *PARP1* expression (*n* = 23) (median OS of 18.6 vs. 41.5 months). (**E**) OSCC patients with low *RAD51* and high *PARP3* expression (*n* = 23) have significantly poorer OS, when compared to OSCC patients with low *RAD51* and low *PARP3* expression (*n* = 23) (median OS of 18.6 vs. 41.5 months). (**F**) OSCC patients with low *PALB2* and high *PARP3* expression (*n* = 23) have significantly poorer OS, when compared to OSCC patients with low *PALB2* and low *PARP3* expression (*n* = 23) (median OS of 18.3 vs. 41.5 months). Statistical analysis was performed using a Kaplan–Meier survival analysis and log-rank (Mantel–Cox) test.

As HR status is a known determinant of response to PARPi, we then assessed the impact of PARP expression on OS in patients with a gene alteration (mutation or CNA) of one of six key HR genes (BRCA1, BRCA2, ATM, RAD51, MRE11, PALB2). In gastric cancer patients with a *BRCA1* gene alteration (mutation or CNA), high *PARP1* expression was demonstrated to be associated with a significantly poorer OS compared to patients with low *PARP1* expression (Figure 6A). This is supported by Liu et al, who demonstrated a significantly poorer OS in gastric cancer patients with high PARP1 protein expression [58]. This suggests that stratification by HR genomic alterations could identify gastric cancer patients who would likely benefit from PARPi. It is important to mention that as mutations and CNAs were grouped together, it is difficult to pinpoint whether mutated or copy number-altered patients demonstrated poorer survival with high PARP expression. Synthetic lethality has been previously demonstrated in gastric cancer pre-clinical studies, with a current trial investigating niraparib in gastric cancer patients stratified by HR status, supporting this approach [96,97,98].

We then assessed the relationship between mRNA expression of the six key HR genes and PARP, and the impact on patient prognosis. The CCA cohort was excluded from this analysis due to an insufficient sample size (Supplementary Materials). Interestingly, OSCC patients with low ATM/RAD51/MRE11/PALB2 expression and high *PARP1*/*2*/*3* expression demonstrated a significantly poorer OS compared to OSCC patients with low *ATM*/*RAD51*/*MRE11*/*PALB2* expression and low *PARP1*/*2*/*3* expression (Figure 6B–F). Hence, stratification by HR mRNA expression identified additional OSCC patients (those with high *PARP1* and *PARP2* expression) who have poorer prognosis with increased DDR PARP expression. This suggests that stratification by HR mRNA expression levels could identify additional patients who may benefit from PARPi.

PAAD patients with low *RAD51*/*MRE11*/*PALB2* expression and high *PARP3*/*4* expression demonstrated a significantly poorer OS compared to PAAD patients with low *RAD51*/*MRE11*/*PALB2* expression and low *PARP3*/*4* expression (Supplementary Materials Figure S1). Hence, no additional patient groups were identified with HR expression stratification. This suggests that using *PARP3*/*4* mRNA expression may be a helpful stratification tool to identify PAAD patients who could gain some benefit from the currently available PARPi, as they contain some PARP3/4 inhibitory action. However, novel potent PARP3/4 inhibitors are required to achieve a potentially greater improvement of the prognosis in PAAD patients.

Our findings on the impact of PARP on prognosis suggest that targeting PARP alone would mostly benefit OSCC, GAC and PAAD patients, especially if stratified by PARP and/or HR status (gene alterations or expression). Therefore, novel targeting approaches with PARPi, such as combination treatment with chemotherapy and/or radiotherapy, may be the best approach for the remaining GI cancer types.

## 9. Conclusions

The four DDR PARP enzymes (PARP1, 2, 3 and 4) have been demonstrated to play various roles in the development and progression of GI cancers. This highlights a potential for the use of PARPi in GI cancers to improve prognosis. Furthermore, the role of DDR PARP enzymes in the repair of cytotoxic therapy-induced DNA damage suggests a role for PARPi in improving therapeutic response to these agents in GI cancers. This is supported by pre-clinical studies, which have demonstrated enhanced therapeutic response with cytotoxic therapy and PARPi combination. This has translated to early phase clinical trials demonstrating promising results, with later phase trials currently investigating the potential benefit of this targeting approach in GI cancers.

Whilst genomic profiling of patient samples in this study demonstrated a low frequency of mutations in DDR PARP and key HR genes, a third of the HR mutations identified are currently being therapeutically exploited by PARPi in other cancer types, suggesting that GI cancer patients with these mutations could potentially benefit from PARPi. Interestingly, we demonstrated that CNA and mRNA expression of the DDR PARP genes is significantly altered in OSCC and OAC cancer types, which could have diagnostic and therapeutic implications and warrants further investigation.

Increased PARP3 and PARP4 mRNA expression was associated with worsened prognosis in OSCC and PAAD patients, suggesting that inhibition of PARP3/4 could have therapeutic benefit in these patients and highlighting the need for the development of PARP3/4-specific PARPi. The mixed results demonstrated from clinical trials of PARPi in GI cancers highlights the need for the identification of biomarkers that can predict efficacy, and thus improve response rates. In this study, stratification of GI cancer patients by HR deficiency (genomically altered or low mRNA expression of key HR genes), identified a subset of GAC and OSCC patients with increased PARP1/2 gene expression and poorer survival, highlighting a potential therapeutic benefit of PARPi in these patients. Whilst further profiling of the protein expression and subcellular location of PARP DDR enzymes and HR biomarkers is required to fully understand the role of PARPi in GI cancer, the literature and data presented here support the potential of PARPi (monotherapy or combination therapy) as a therapeutic strategy in GI cancers.

## Figures and Tables

**Table 1:** Ongoing clinical trials assessing the combination of PARP inhibitors with chemoradiotherapy in GI cancers.

Phase	PARP Inhibitor	Combination Therapy	Cancer Type	Trial Number
**I**	Talazoparib	Trifluridine/Tipiracil	Gastroesophageal Adenocarcinoma, Colorectal Cancer	NCT04511039
**I**	Fluzoparib	Paclitaxel + Apatinib	Gastroesophageal Adenocarcinoma	NCT03026881
**I**	Fluzoparib	FOLFIRINOX	Resectable Pancreatic Cancer	NCT04425876
**I**/**II**	Fluzoparib	FOLFIRINOX	Advanced Pancreatic Cancer	NCT04228601
**I**/**II**	Olaparib	Oxaliplatin + Tegafur/Gimeracil Oteracil Potassium	Gastric Cancer	NCT04410887
**I**/**II**	Veliparib	FOLFOX	Pancreatic Cancer	NCT01489865
**I**/**II**	Rucaparib	Irinotecan, Leucovorin, Fluorouracil	Gastrointestinal Malignancies	NCT03337087
**II**	Olaparib	Temozolomide	MGMT-Hypermethylated Colorectal Cancer	NCT04166435
**II**	Olaparib	Paclitaxel + Pembrolizumab	Gastric Cancer	NCT04209686
**II**	Olaparib	Paclitaxel + Durvalumab	Gastric Cancer	NCT03579784
**II**	Olaparib	Paclitaxel	Gastric Cancer	NCT01063517
**II**	Veliparib	Gemcitabine + Cisplatin	BRCA/PALB2-mutated Pancreatic Cancer	NCT01585805
**II**	Veliparib	FOLFIRI	Pancreatic Cancer	NCT02890355

**Table 2:** Frequency of PARP Mutations and CNA in the GI Cancer Cohorts—represents zero mutations/CNA; *n*, total number of patients. Frequency (%) was calculated as the percentage of patients who had a gene mutation/CNA from the total number of patients in the cohort of the same cancer type.

PARP Gene Alteration	Genomic Alteration Frequency (%)								
	OAC	OSCC	GAC	HCC	CCA	PAAD	COAD	READ	Total
	*n* = 87	*n* = 95	*n* = 440	*n* = 369	*n* = 36	*n* = 184	*n* = 378	*n* = 155	*n* = 1744
**Mutation**									
**PARP1**	1.1	1.1	2.7	0.5	-	1.1	2.1	0.6	1.5
**PARP2**	-	-	1.8	0.3	-	0.5	0.8	0.6	0.8
**PARP3**	2.3	-	1.6	-	-	-	1.9	-	0.9
**PARP4**	2.3	2.1	4.3	0.8	-	-	3.2	1.3	2.3
**CNA**									
**PARP1**	1.1	2.1	2.0	5.1	5.6	2.2	0.5	0.6	2.3
**PARP2**	1.1	3.2	1.6	0.8	-	0.5	-	0.6	0.9
**PARP3**	3.4	2.1	0.9	0.3	-	-	0.3	0.6	0.7
**PARP4**	4.6	1.1	0.9	0.8	-	0.5	4.5	4.5	2.1

**Table 3:** Frequency of HR Mutations and Copy Number Alterations Across the Gastrointestinal Cancer Cohorts. HR, homologous recombination—represents zero mutations/CNA; *n*, total number of patients. Frequency (%) was calculated as the percentage of patients who had a gene mutation/CNA from the total number of patients in the cohort of the same cancer type.

HR Gene Alteration	Genomic Alteration Frequency (%)								
	OAC	OSCC	GAC	HCC	CCA	PAAD	COAD	READ	Total
	*n* = 87	*n* = 95	*n* = 440	*n* = 369	*n* = 36	*n* = 184	*n* = 378	*n* = 155	*n* = 1744
**Mutation**									
**BRCA1**	1.1	1.1	3.6	1.4	-	1.1	2.9	1.9	2.2
**BRCA2**	3.4	4.2	8.9	2.2	2.8	1.1	5.8	5.2	5.0
**ATM**	6.9	3.2	10.5	3.5	2.8	4.3	9.8	9.0	7.3
**RAD51**	-	-	0.5	0.5	-	-	-	-	0.2
**MRE11**	-	-	1.6	-	-	0.5	1.1	1.9	0.9
**PALB2**	-	-	2.5	0.3	-	0.5	1.9	1.3	1.3
**CNA**									
**BRCA1**	3.4	-	2.3	1.6	-	2.2	0.3	0.6	1.4
**BRCA2**	6.9	1.1	3.2	0.8	-	-	2.6	3.9	2.3
**ATM**	2.3	-	2.3	0.8	-	-	0.5	0.6	1.0
**RAD51**	1.1	1.1	0.2	0.3	-	0.5	1.9	1.3	0.8
**MRE11**	1.1	2.1	1.6	0.5	-	-	0.3	-	0.7
**PALB2**	-	-	0.5	-	-	-	1.1	-	0.3

## Data Availability

The data presented in this study are openly available at The Cancer Genome Atlas (TCGA) consortium (PanCancer Atlas), at doi:10.1038/nature13480, 10.1016/j.cell.2017.05.046, 10.1038/nature11252, 10.1038/nature20805, 10.1016/j.cell.2018.02.052 [181,182,183,184,185]; Oncology Knowledge Base (OncoKB) at doi:10.1200/PO.17.00011 [186].

## Supplementary Materials

The following are available online at https://www.mdpi.com/article/10.3390/biomedicines9081024/s1, Method S1: Patient and Gene Data Collection, Method S2: Gene Data Measurement & Analysis, Method S3: Survival Analysis, Method S4: Statistical Analysis, Figure S1: Effect of DDR PARP gene expression on overall survival outcomes of PAAD patients with low HR gene expression.
