# Supplementary material for: Therapeutic Potential of PARP Inhibitors in the Treatment of Gastrointestinal Cancers
Source: Biomedicines. 2021 Aug 16;9(8):1024. doi: 10.3390/biomedicines9081024 (PMC8392860; doi:10.3390/biomedicines9081024)
Supplement: Supplementary file 1 [file biomedicines-09-01024-s001.zip › biomedicines-1309392-supplementary.pdf]

biomedicines

MDPI

Supplementary Material Methods

Patient and Gene Data Collection

Patients were selected from The Cancer Genome Atlas (TCGA) consortium (PanCancer Atlas), publicly available data sets of 1,744 gastrointestinal cancer patients (87 oesophageal Adenocarcinoma (OAC), 95 oesophageal squamous cell carcinoma (OSCC), 440 gastric adenocarcinoma (GAC), 369 hepatocellular carcinoma (HCC), 36 cholangiocarcinoma (CCA), 184 pancreatic adenocarcinoma (PAAD), 378 colon adenocarcinoma (COAD) and 155 rectal adenocarcinoma patients (READ)) [179-183]. The cbioportal genomic data visualization platform (www.cbioportal.org) was used to locate and download data on the mutations, copy number alterations (CNA) and mRNA expression of 4 PARP (PARP1, PARP2, PARP3, PARP4) and 6 HR genes (BRCA1, BRCA2, ATM, RAD51 MRE11, PALB2) of the GI cancer patients from the TCGA consortium [186, 187]. Additionally, the survival data of the patients was obtained from the TCGA consortium using the cbioportal platform.

Gene Data Measurement & Analysis

The frequency of mutations or CNA were calculated as the percentage of patients who had a mutation/CNA in the gene from the total number of patients in the cohort of the same cancer type. Gene Copy Numbers were classified based on the Genomic Identification of Significant Targets in Cancer (GISTIC) algorithm **[186,187]**]. mRNA expression was presented as RSEM, as transcript quantification was obtained from the RNA-sequencing data using the RSEM (RNA-Seq by Expectation Maximization) program [185]. The oncogenic and therapeutic implications of mutations and CNAs were identified from the Oncology Knowledge Base (OncoKB). OncoKB is a precision oncology knowledgebase that curates information on the effects and therapeutic implications of specific gene alterations [184].

Survival Analysis

The survival analysis was performed using a Kaplan-Meier survival analysis. Patients that did not have an event occurrence (death) were censored. For cohorts that did not reach a 50% event occurrence (death) rate, the data was not sufficient to calculate their median OS and it was classified as 'undefined'. The median expression was used to stratify patients into high versus low expression. Patients with expression values higher than the median were placed in the 'high expression' group, while patients with expression values lower than the median were placed in the 'low expression' group. Cohorts without at least 10 patients in each arm were excluded from the survival analysis.

Statistical Analysis

Statistical analysis was carried out using GraphPad Prism version 8.4.3 (GraphPad Software Inc). Data are presented as mean ± the standard error of the mean (SEM). Statistical analysis of categorical data was performed using a one-sided Fisher's exact test. Statistical analysis of continuous numerical data was performed using a two-tailed Student's *t*-test, one-way ANOVA with Tukey's multiple comparison *post-hoc* test or Mann Whitney U non-parametrical testing. Testing for normal distribution of the data was performed by a D'Agostino & Pearson test. Survival analysis was performed using a Kaplan-Meier survival analysis and statistical significance was determined by a log-rank (Mantel-Cox) test. For all statistical analyses, differences were considered to be statistically significant at $p < 0.05$.

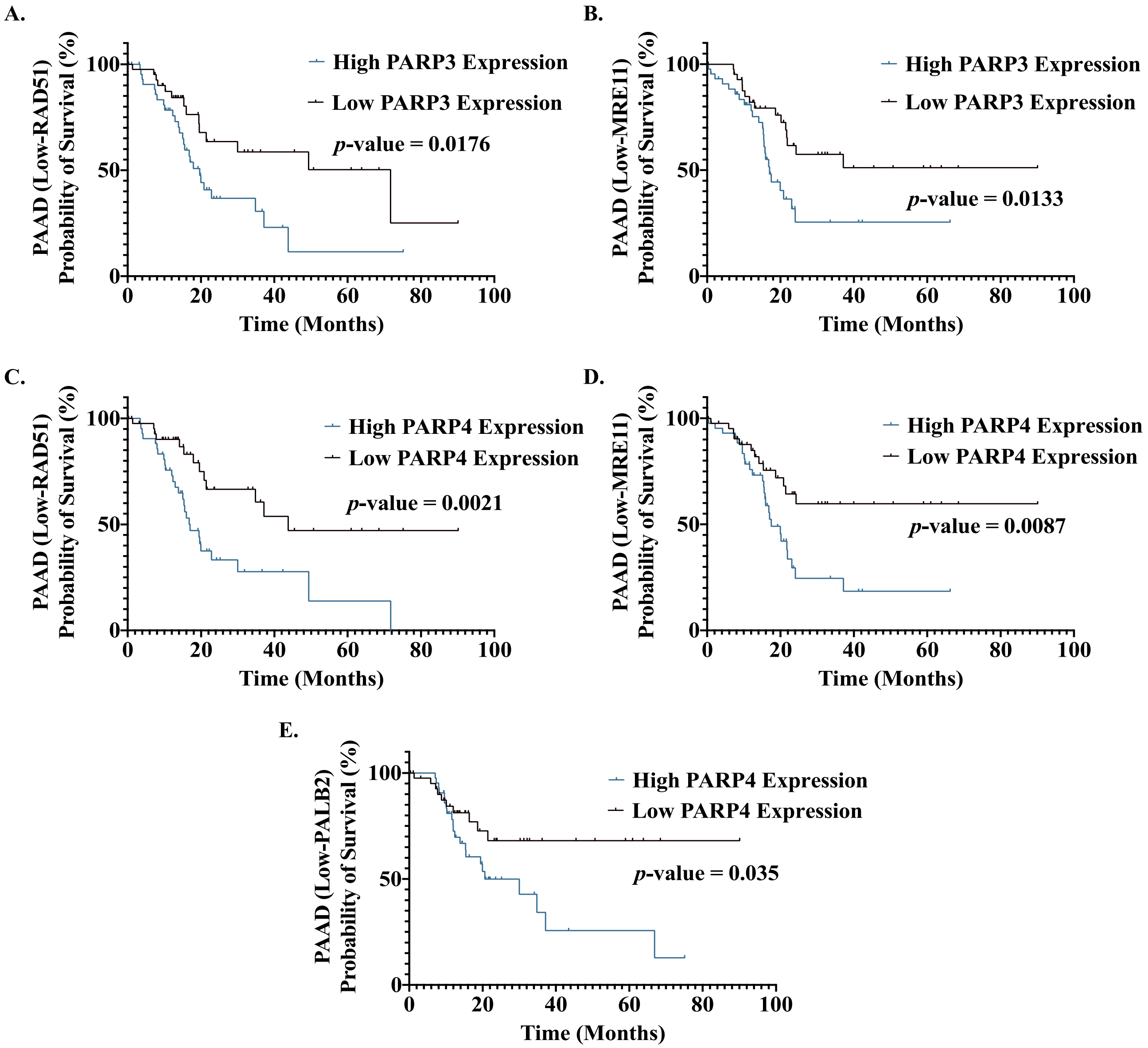

Supplementary Figure S1. Effect of DDR PARP gene expression on overall survival outcomes of PAAD patients with low HR gene expression. A. PAAD patients with low *RAD51* and high *PARP3* expression (*n*=44) have significantly poorer OS, when compared to PAAD patients with low *RAD51* and low *PARP3* expression (*n*=44) (Median OS 19.7 vs. 71.7 months, *p*=0.0176) B. PAAD patients with low *MRE11* and high *PARP3* expression (*n*=44) have significantly poorer OS, when compared to PAAD patients with low *MRE11* and low *PARP3* expression (*n*=44) (Median OS 17 months vs. undefined, *p*=0.0133) C. PAAD patients with low *RAD51* and high *PARP4* expression (*n*=44) have significantly poorer OS, when compared to PAAD patients with low *RAD51* and low *PARP4* expression (*n*=44) (Median OS 17 vs. 43.8 months, *p*=0.0021) D. PAAD patients with low *MRE11* and high *PARP4* expression (*n*=44) have significantly poorer OS, when compared to PAAD patients with low *MRE11* and low *PARP4* expression (*n*=44) (Median OS 17.5 months vs. undefined, *p*=0.0087) E. PAAD patients with low *PALB2* and high *PARP4* expression (*n*=44) have significantly poorer OS, when compared to PAAD patients with low *PALB2* and low *PARP4* expression (*n*=44) (Median OS 20.6 months vs. undefined, *p*=0.035).Statistical analysis was performed using a Kaplan-Meier survival analysis and log-rank (Mantel-Cox) test.
